# Supplementary material for: NLRP7 Promotes Choriocarcinoma Growth and Progression through the Establishment of an Immunosuppressive Microenvironment
Source: Cancers (Basel). 2021 Jun 15;13(12):2999. doi: 10.3390/cancers13122999 (PMC8232770; doi:10.3390/cancers13122999)
Supplement: Supplementary file 1 [file cancers-13-02999-s001.zip › cancers-1233085-supplementary.pdf]

Supplementary Materials: NLRP7 Promotes Choriocarcinoma Growth and Progression through the Establishment of an Immunosuppressive Microenvironment

Deborah Reynaud, Roland Abi Nahed, Nicolas Lemaitre, Pierre-Adrien Bolze, Wael Traboulsi, Frederic Sergent, Christophe Battail, Odile Filhol, Vincent Sapin, Houssine Boufettal, Pascale Hoffmann, Touria Aboussaouira, Padma Murthi, Rima Slim, Mohamed Benharouga and Nadia Alfaidy

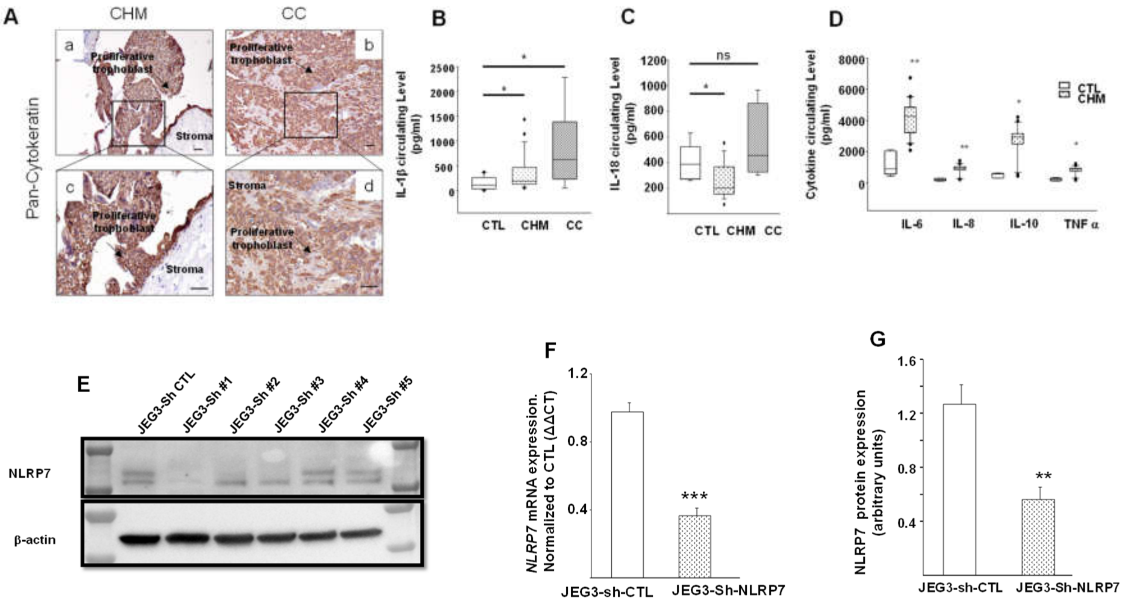

Figure S1. Cytokeratin staining's, comparison of circulating cytokines in control, CHM and CC patients and comparison of NLRP7 expression upon Sh inactivation Strategy. Panel A reports CHM and CC placenta sections that were stained with pan-cytokeratin to identify trophoblast cells. Microphotographs in c and d are higher magnifications of microphotographs in a and b, respectively. Panel B reports IL-1β circulating levels in normal pregnant women (CTL, n=9); CHM (n=12) and CC patients (n=9) during the first trimester of pregnancy. Panel C reports a graph that compares the levels of IL-18 in the same cohorts. Panel D reports a graph that compares the levels of IL-6, IL-8, IL-10 and TNFα levels in CTL and CHM patients. Data are presented as box plots. * $p < 0.05$, ** $p < 0.01$; ns: non-significant. Panel E reports a Western blot analysis that compares the levels of expression of NLRP7 between the five cell lines of JEG3-Sh-NLRP7. Standardization of protein signals was performed using antibodies against β-actin. Panel F compares the levels of *NLRP7* mRNA expression between JEG3-Sh-CTL and JEG3-Sh-1NLRP7 (n=5 independent experiments). Three control genes, *RPL0*, *18S* rRNA and *GAPDH* were used to standardize for mRNA expression. The $2^{-\Delta\Delta Ct}$ methodology was employed. Panel G reports a quantification of the protein levels of NLRP7 in JEG3-Sh-CTL and JEG3-Sh-1-NLRP7. Four independent experiments were performed Data are presented mean ± SEM, ** $p < 0.01$ *** $p < 0.001$.

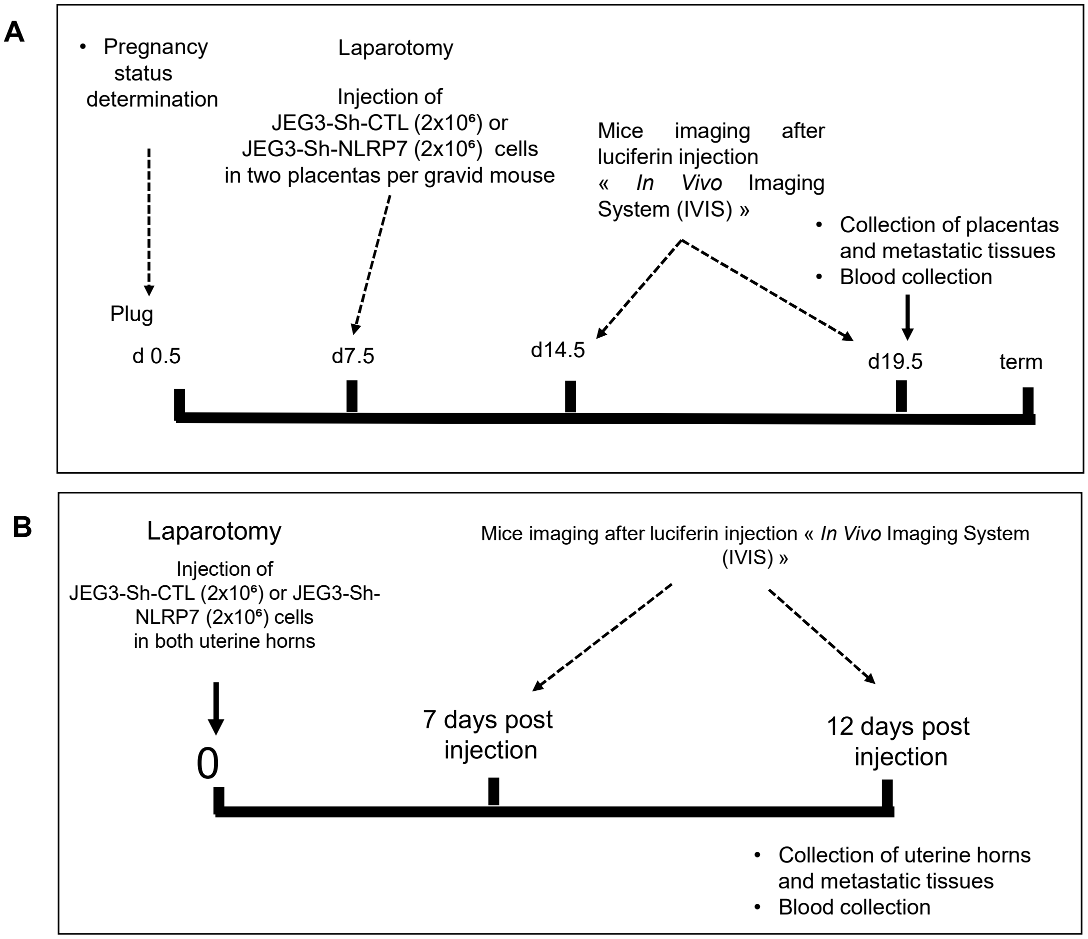

Figure S2. Comparison of circulating cytokines in control, CHM and CC patients and comparison of NLRP7 expression upon Sh inactivation Strategy. Panel A illustrates the flow chart of the experimental procedure of the orthotopic model. The gravid mice were randomly assigned to be injected on day 7.5 of gestation either with JEG3-Sh-CTL (n=8) or in JEG3-Sh-NLRP7 ($n = 7$). Mice were imaged at day 14.5 and 19.5 dpc of gestation using IVIS imaging system and sacrificed at 19.5 dpc. Placenta, metastatic tissues and maternal blood were collected following imaging. Panel B illustrates the flow chart of the experimental procedure used for non-gravid mice injected in the uterine horns. The mice were randomly assigned to be injected either with JEG3-Sh-CTL ($n = 3$) or in JEG3-Sh-NLRP7 ($n = 3$). Mice were imaged at 7 and 12 days post injection (dpi) using IVIS imaging system and sacrificed at 12 dpi. Tissues and blood were collected after imaging.

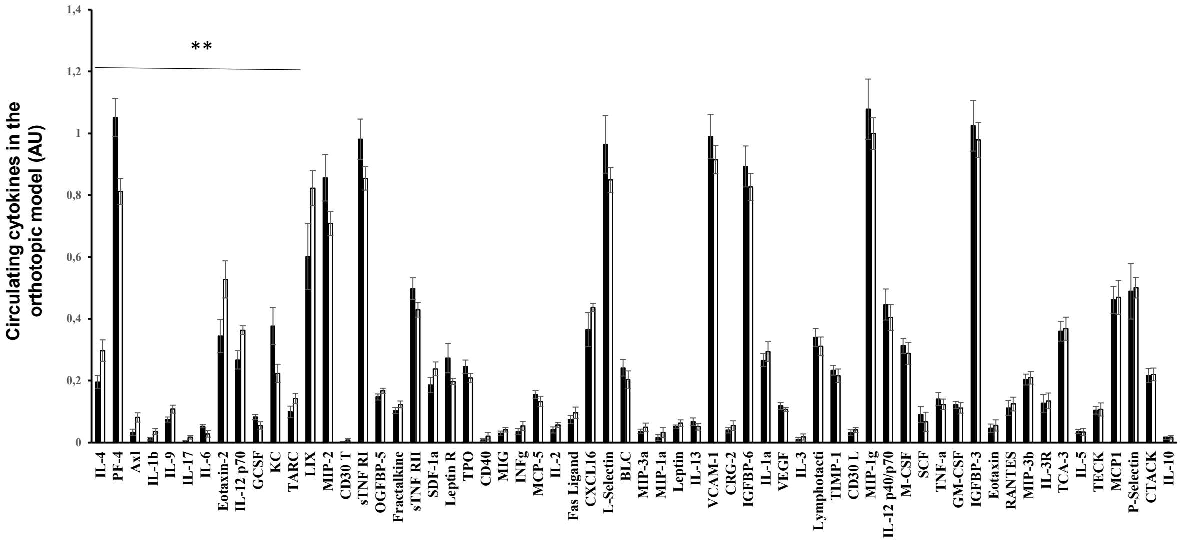

Figure S3. Circulating cytokine levels in mice injected with JEG3-Sh-CTL or JEG3-Sh-NLRP7 cells. Panel A reports the global analysis of the 62 inflammatory cytokines. Measurement of the levels of these cytokines was assessed in sera collected from mice injected with JEG3-Sh-NLRP7 and JEG3-Sh-CTL in the placenta (orthotopic model). Detailed information's about the significantly differentiated cytokines are reported in Figure 7D. Data are represented as mean ± SEM. * $p < 0.05$.

Figure 1I

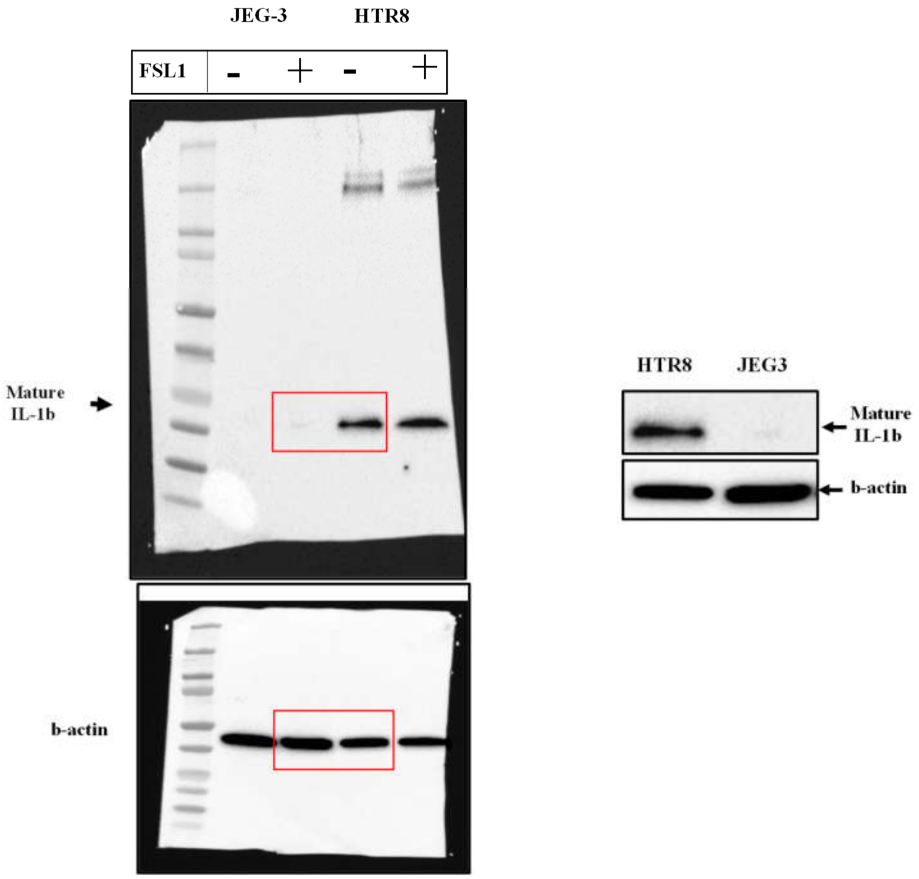

Figure II

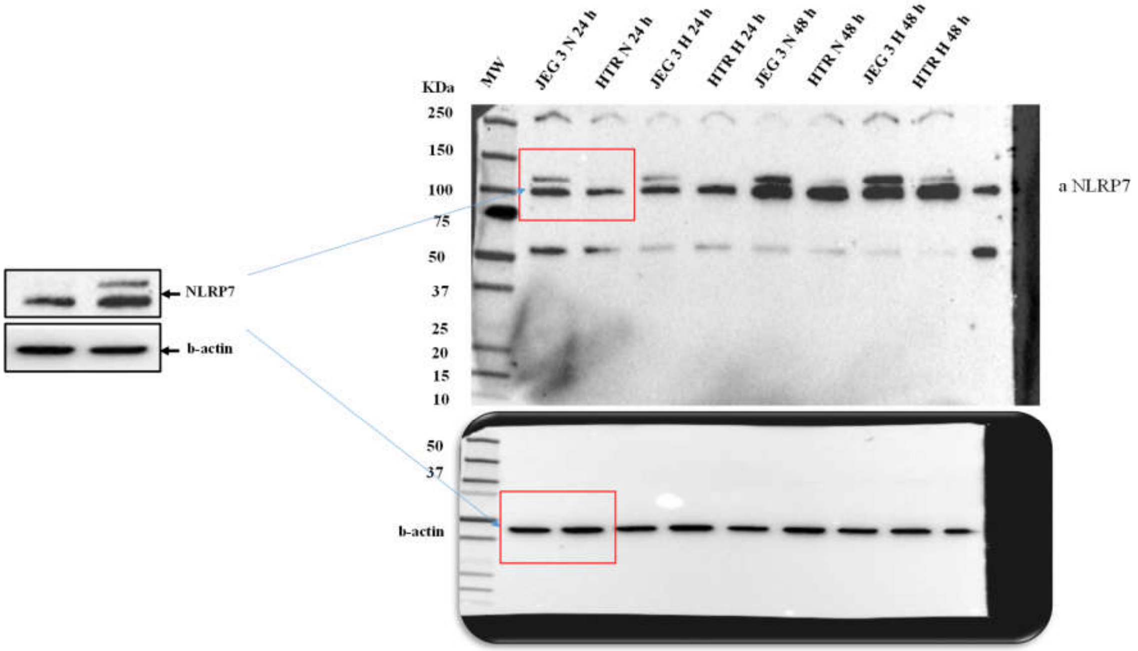

PCNA western blotting

Figure 3 E

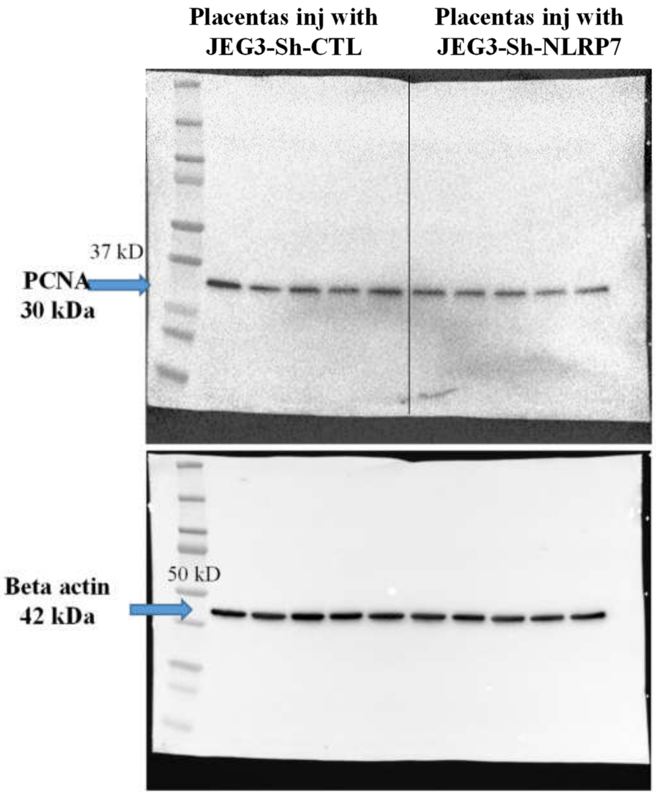

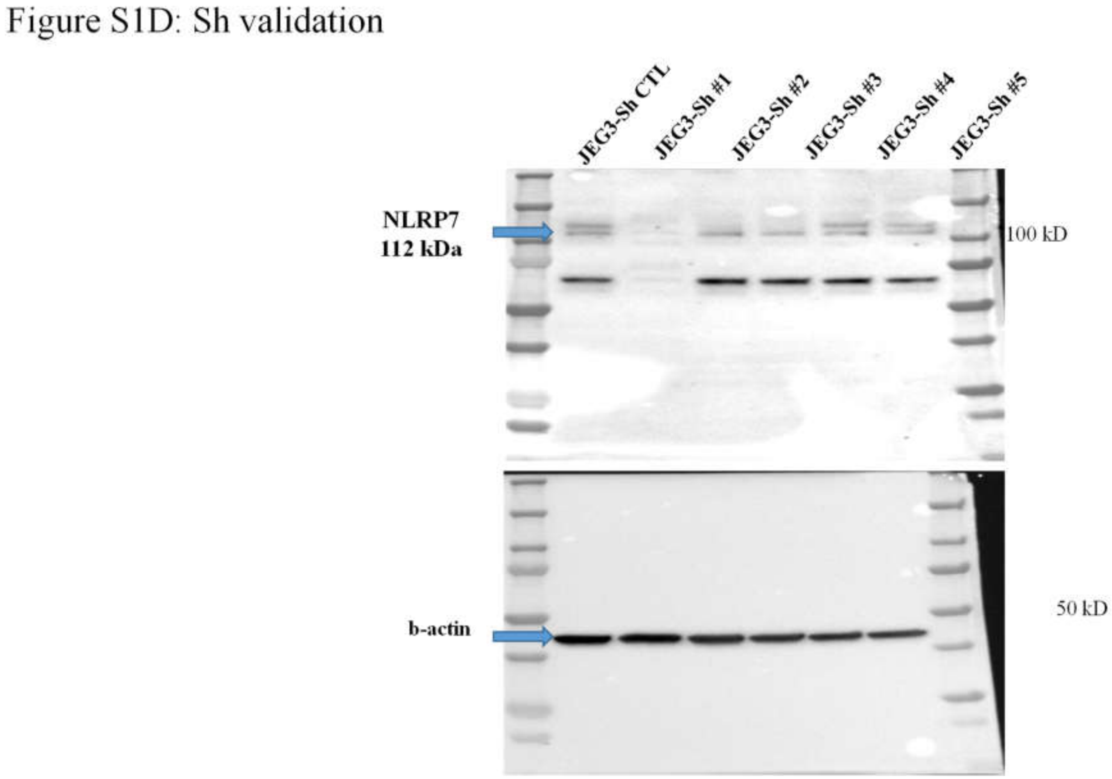

Figure s4. Original Western Blots.

Table S1. Clinical characteristics of normal and pathological pregnancies.

Maternal Characteristics	Normal Gestation	Complete Hydatidiform Mole	Choriocarcinoma
Number of samples	N = 29	N = 26	N = 11
Maternal age	28,6 (18–50)	37,5 (19–53)	36,6 (21–43)
Gestational age at sampling	10 (9–12)	11 (8–16)	11 (9–12)
◎hCG at sampling (IU/L)	62054 (4560–262379)	490701 (57151–1480000)	338202 (10406–1077379)
Parity	0-2	0-5	0-3

Table S2. Primers used for the RT-qPCR analyses.

**Primers Used for the RT-qPCR**			
**Gene**	**Primer Forward**	**Primer Reverse**	**Temperature (°C)**
NLRP7	TGCTGTACAAGACCATGACACG	ACTCAAGCCCTCACACAGAAAC	60
ASC	TGGATGCTCTGTACGGGAAG	CCAGGCTGGTGTGAAACTGAA	60
CASP1	TGCCTGTTCCTGTGATGTGG	TGTCCTGGGAAGAGGTAGAAACATC	60
IL-1b	GTCGGAGATTCGTAGCTGGAT	GTCGGAGATTCGTAGCTGGAT	60
IL-18	TGCCAACTCTGGCTGCTAAA	TTGTTGCGAGAGGAAGCGAT	60
18S	AAACGGCTACCACATCCAAG	CCTCCAATGGATCCTCGTTA	60

RPL0	TCGACAATGGCAGCATCTAC	ATCCGTCTCCACAGACAAGG	60
GAPDH	ACCCAGAAGACTGTGGATGG	TTCTAGACGGCAGGTCAGGT	60
